# L-Ilf3 and L-NF90 Traffic to the Nucleolus Granular Component: Alternatively-Spliced Exon 3 Encodes a Nucleolar Localization Motif

**DOI:** 10.1371/journal.pone.0022296

**Published:** 2011-07-19

**Authors:** Wildriss Viranaicken, Laila Gasmi, Alexandre Chaumet, Christiane Durieux, Virginie Georget, Philippe Denoulet, Jean-Christophe Larcher

**Affiliations:** 1 UPMC Univ Paris 06, UMR 7622, Laboratoire de Biologie du Développement, Paris, France; 2 CNRS, UMR 7622, Laboratoire de Biologie du Développement, Paris, France; 3 Institut Jacques Monod, UMR7592 CNRS - Université Denis Diderot, Paris, France; 4 UPMC Université Paris 06, IFR 83, Institut de Biologie Intégrative, Paris, France; Ludwig-Maximilians-Universität München, Germany

## Abstract

Ilf3 and NF90, two proteins containing double-stranded RNA-binding domains, are generated by alternative splicing and involved in several functions. Their heterogeneity results from posttranscriptional and posttranslational modifications. Alternative splicing of exon 3, coding for a 13 aa N-terminal motif, generates for each protein a long and short isoforms. Subcellular fractionation and localization of recombinant proteins showed that this motif acts as a nucleolar localization signal. Deletion and substitution mutants identified four arginines, essential for nucleolar targeting, and three histidines to stabilize the proteins within the nucleolus. The short isoforms are never found in the nucleoli, whereas the long isoforms are present in the nucleoplasm and the nucleoli. For Ilf3, only the posttranslationally-unmodified long isoform is nucleolar, suggesting that this nucleolar targeting is abrogated by posttranslational modifications. Confocal microscopy and FRAP experiments have shown that the long Ilf3 isoform localizes to the granular component of the nucleolus, and that L-Ilf3 and L-NF90 exchange rapidly between nucleoli. The presence of this 13 aminoacid motif, combined with posttranslational modifications, is responsible for the differences in Ilf3 and NF90 isoforms subcellular localizations. The protein polymorphism of Ilf3/NF90 and the various subcellular localizations of their isoforms may partially explain the various functions previously reported for these proteins.

## Introduction

Compartmentalization of proteins is a key mechanism for regulating many cellular processes and/or restricting site activity of proteins. To find their right place in cells, proteins are generally endowed with signals that target them to the appropriate subcellular compartment. This destination can represent either the final working place of proteins, a transient localization or a means for certain other proteins to be sequestered. These signals are recognized and processed by specialized cell machineries. Another way to localize proteins is to first direct their mRNA prior to their translation, a mechanism requiring specific nucleotide signals as well as escort proteins to process these signals.

In this context, we previously characterized two proteins, Interleukin enhancer binding factor 3 (Ilf3) and Nuclear Factor 90 (NF90), that interact with the axonal targeting element of Tau mRNA and move with it from the nucleus to the axon hillock [1]. Belonging to the family of proteins containing double-stranded RNA-binding domain(s) (dsRBM; [2]), these two proteins are generated by alternative splicing from a single gene [3], [4]. This event provides two proteins with common N-terminal and central domains and a specific C-terminal domain [3]–[5].

An heterogeneity of Ilf3 and NF90 was evidenced after separation by 2-D PAGE [1] with at least 12 and 8 spots, respectively. This polymorphism is partially due to an alternative splicing of exon 3 in the 5′ region of their premessenger RNA, which generates long (L) and short (S) isoforms for Ilf3 and NF90 [5]. These isoforms differ by the presence or the absence of a specific basic N-terminal sequence of 13 residues (ALYHHHFITRRRR) localized just downstream the initiation methionine. The polymorphism of Ilf3 and NF90 arising from alternative splicing events is also complexified by at least two posttranslational modifications: arginine-methylation by protein-arginine methyltransferase I in the RGG motif [6] and phosphorylation by PKR [4], [7], [8], the DNA protein kinase [9] or the AKT kinase in T-cells [10].

The existence of several Ilf3 and NF90 isoforms [1], [3], [5] may reflect their numerous described functions: transcriptional activation [11]–[14], eukaryotic and viral RNA binding [1], [15]–[20], translational inhibition [21]–[24] or enzymatic regulation [6], [7]. This polymorphism could also explain the various subcellular localizations described [1], [25], [26]. Since Ilf3 and NF90 are recovered in both nuclear and cytoplasmic fractions [1], they may shuttle between these compartments [27]. Finally, the interaction of Ilf3 and NF90 with protein and/or RNA partners may be regulated by posttranscriptional and/or posttranslational modifications [13], [23].

Following our original report of an additional splicing event that generates multiple Ilf3 and NF90 protein profiles isoforms [5], we herein investigated the potential influence of Ilf3 and NF90 posttranscriptional and posttranslational heterogeneities on their subcellular localization. The N-terminal location of the alternative 13-aa segment enriched in basic residues led us to search for a specific role to this signal-type motif. Accordingly, we fused the N-terminal sequence of Ilf3/NF90, containing or not the 13 residues, to GFP or a well-known cytoplasmic protein to follow whether the chimeric proteins were targeted to a specific cellular compartment. Since some chimeric proteins were targeted in the nucleolus, we performed subcellular fractionation to confirm the nucleolar localization of endogenous proteins. In addition, using deletion and substitution mutants, we investigated on the role played by different residues present in this motif. Finally, confocal microscopy and FRAP experiments were performed to study the accurate subnucleolar localization and the dynamics of the proteins.

## Results

### 1. Long Ilf3/NF90 N-terminus fused with GFP accumulates in nuclear foci

Whereas L- and S-Ilf3/NF90 isoforms exhibit the same ability to bind Tau mRNA [1], we tested whether the L-isoforms containing the specific N-terminal sequence exhibited a particular localization. Plasmids containing the GFP sequence fused in frame behind the sequences coding for the 54 first aa of the L-Ilf3/NF90 isoforms (Long-GFP) or the 41 aa of the S-Ilf3/NF90 isoforms (Short-GFP) were transfected into HeLa cells. The intracellular distribution of GFP was observed by epifluorescence microscopy.

**Figure 1:**
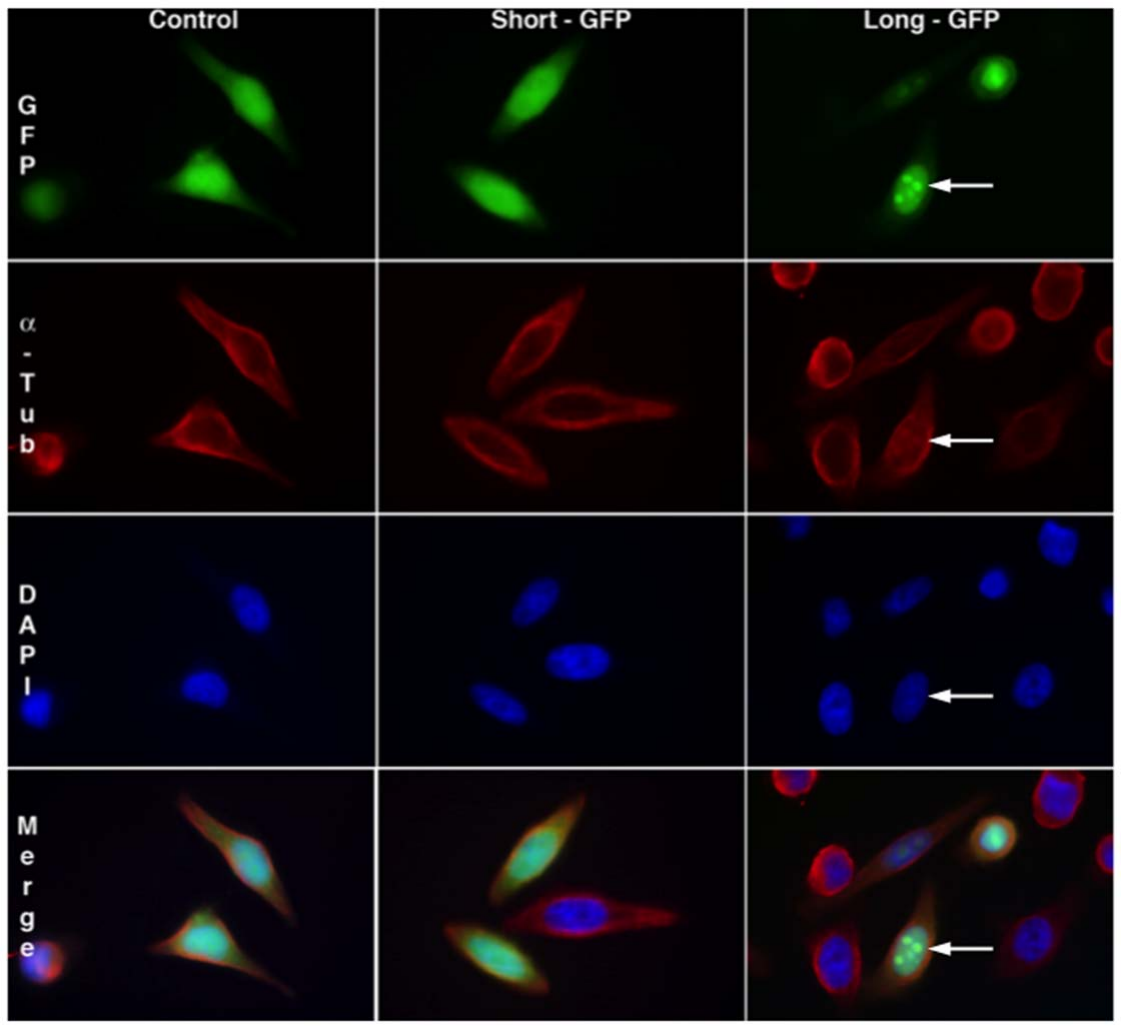
Subcellular localization of GFP fused with the short or long N-terminal sequence of Ilf3/NF90. Plasmids pEGFP-N1 (Control, left panels), pEGFP-N1-Ilf3/NF90 common N-terminal short sequence (Short-GFP, mid panels) and pEGFP-N1-Ilf3/NF90 common N-terminal long sequence (Long-GFP, right panels) were transfected into HeLa cells. After 24 hours, cells were fixed and co-stained with anti-α-tubulin antibody (α-Tub) and DAPI. GFP or GFP fusion proteins appear in green, α-tubulin in red and DAPI staining in blue. Arrows point to intranuclear foci.

In control cells expressing GFP alone as in cells transfected with the Short-GFP plasmids, fluorescence was observed in the cytoplasm and especially in the nucleus, where GFP has been previously shown to accumulate (Figure 1, left and middle panels). In contrast, fluorescence was barely detectable in the cytoplasm of cells transfected with the Long-GFP plasmids and rather accumulated in discrete nuclear foci that may correspond to nucleoli (Figure 1, right panels). This 13-aa sequence could thus function as an efficient nucleolar localization signal (NoLS).

### 2. Endogenous long Ilf3/NF90 isoforms are targeted to the nucleolus

**Figure 2:**
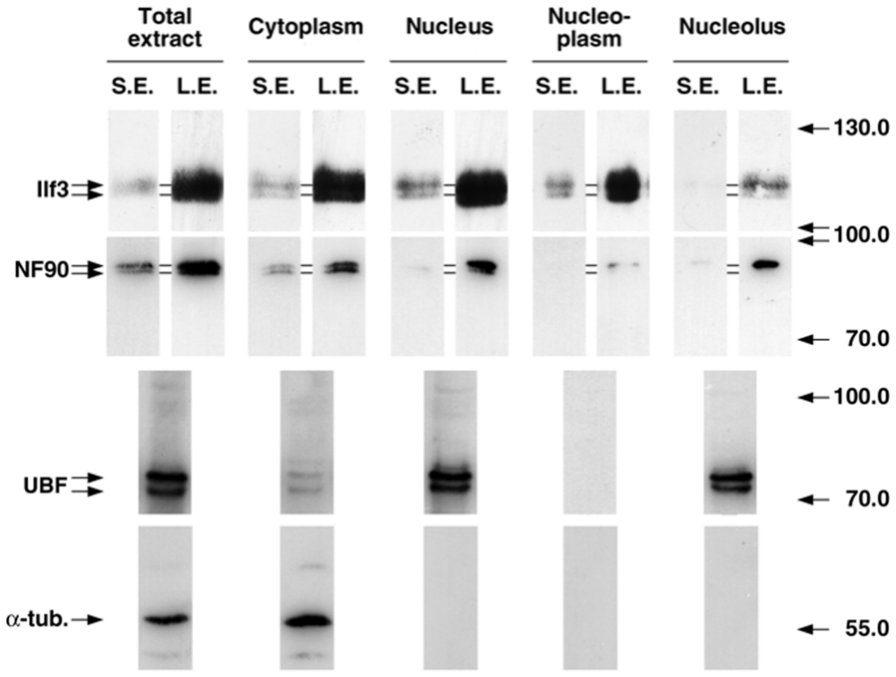
Subcellular distribution of Ilf3 and NF90 in P19 cells. After subcellular fractionation, proteins from identical percentages of each fraction were submitted to SDS-PAGE, blotted onto nitrocellulose and immunodetected with the serum Ab78 raised against Ilf3 and NF90 (S.E.: short exposure time; L.E.: long exposure time), anti-UBF serum (UBF) or anti-α-tubulin antibody (α-tub.). Molecular weight markers (kDa) are indicated at the right.

To confirm the role of the 13-aa motif in nucleolar targeting and to avoid any bias associated with overexpression, the presence of endogenous L- and S-Ilf3/NF90 isoforms was examined in subcellular fractions prepared from P19 cells. Identical results were obtained with HeLa cells (data not shown). Western blotting of different fractions using a polyclonal antibody raised against common regions of the two proteins [1] revealed that the L- and S-Ilf3 isoforms were present in both cytoplasmic and nuclear fractions (Figure 2). In contrast, only the L-NF90 isoforms were found in the nuclear fraction whereas all the NF90 isoforms were recovered in the cytoplasm. Furthermore, when purified nuclei were fractionated into nucleoplasmic and nucleolar fractions, only the L-Ilf3 and L-NF90 isoforms were found associated with the nucleoli. The identity of the different fractions was checked by immunodetecting UBF, as a nucleolar marker, and α-tubulin, as a cytosolic marker. These results strengthen the idea that the N-terminal 13-aa sequence present in the L-Ilf3 and L-NF90 isoforms acts as a potent NoLS.

**Figure 3:**
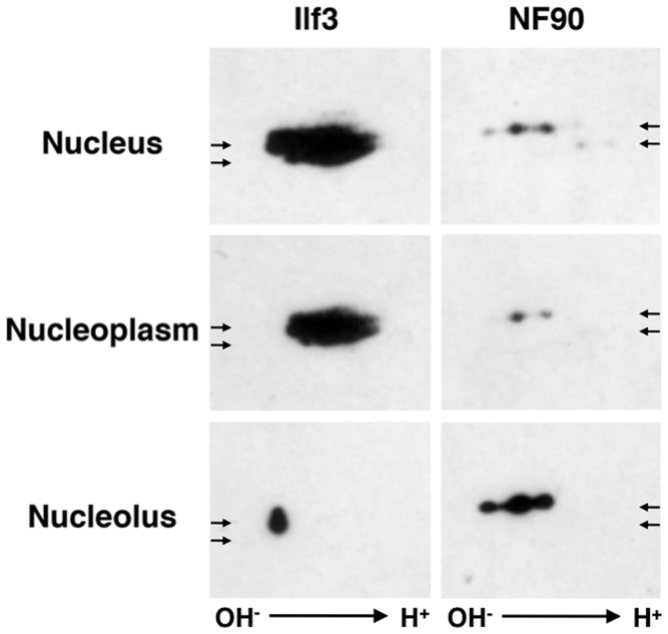
Ilf3 and NF90 polymorphism in nuclear fractions purified from P19 cells. Ilf3 and NF90 from P19 cell nuclear fractions were submitted to 2-D PAGE and immunodetected with polyclonal antibody 78. Arrows positioned in the same coordinates in Ilf3 or NF90 panels indicate the positions of Ilf3 and NF90 long and short isoforms. The faint signal in the middle right panel was detected with a ten fold longer time exposure than that of the other panels.

To confirm the targeting of L-isoforms to the nucleolus and explore the role of their posttranslational modifications in this compartimentalization, subcellular fractions were analyzed by 2D-PAGE. For isoforms present in both cytosolic and nuclear fractions (L- and S-Ilf3 and L-NF90), the same 2-D patterns were obtained (data not shown), suggesting that the posttranslational modifications do not regulate their nucleocytoplasmic distribution. However, the comparison of the Ilf3 and NF90 heterogeneities in subnuclear compartments showed an exclusive distribution of the isoforms between the nucleolus and the nucleoplasm (Figure 3). As shown above (Figure 2), the nuclear NF90 isoforms were almost solely recovered in the nucleolar fraction (Figure 3, lower right panel). The faint signal observed in the nucleoplasmic fraction (Figure 3, middle right panel) was detected with a ten-fold longer exposure time than that obtained with the nucleolar extract and is probably due to L-NF90 isoforms trafficking between the nucleolus and the cytosol. Concerning Ilf3, only the most alkaline L-isoforms were exclusively recovered in the nucleolar fraction whereas all the other L- and S-isoforms were found in the nucleoplasm. These results confirm that the nucleolar fraction contains only isoforms of Ilf3/NF90 containing the 13 aminoacid motif and strongly suggest that posttranslational modifications of L-Ilf3 isoforms, evidenced by their different more acidic pI, negatively regulate their nucleolar localization.

### 3. A cytoplasmic protein fused to Long Ilf3/NF90 N-terminus is targeted to the nucleolus

**Figure 4:**
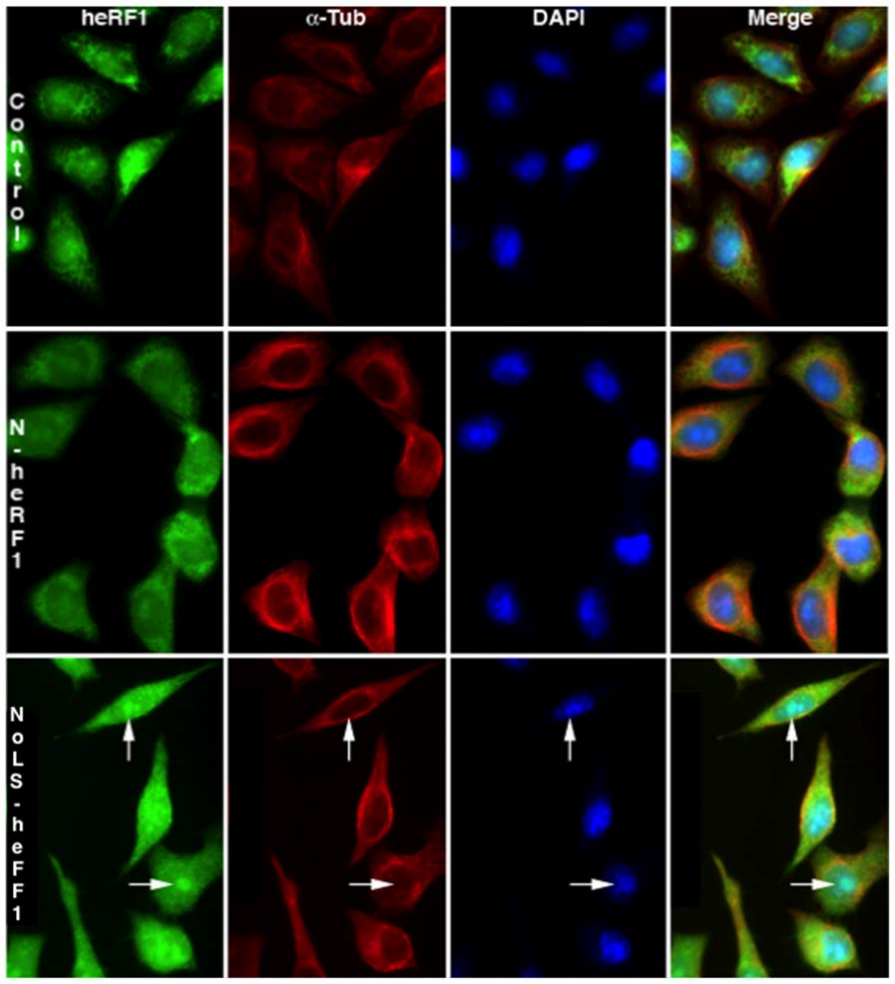
Subcellular localization of human eRF1 fused with short or long N-terminal sequence of Ilf3/NF90. Plasmids pCMV-heRF1-Ilf3/NF90 short N-terminal sequence (N-heRF1, mid panels) and pCMV-heRF1-Ilf3/NF90 long N-terminal sequence (NoLS-heRF1, lower panels) were transfected into HeLa cells. After 24 hours, untransfected (Control, upper panels) or transfected cells were co-stained with anti-heRF1 antibody (heRF1), anti-α-tubulin antibody (α-Tub) and DAPI. Endogenous heRF1 or heRF1 recombinant fusion proteins appear in green, α-tubulin in red and DAPI in blue. Arrows point to intranuclear foci corresponding to nucleoli.

Since GFP can enter the nucleus, we tested the ability of the 13-aa motif to target to the nucleus and nucleolus a well-known cytoplasmic protein, the human eukaryotic releasing factor 1 (heRF1). The heRF1 coding sequence was fused at the 3′ end of the sequence coding for the first 54 aa of the L-Ilf3/NF90 isoforms (NoLS-heRF1) or for the first 41 aa of the S-Ilf3/NF90 isoforms (N-heRF1). The constructs were transfected into HeLa cells and the heRF1 localization was observed by immunofluorescence microscopy (Figure 4).

In control cells transfected with N-heRF1 (Figure 4, middle panels), anti-eRF1 antibodies stained only the cytoplasmic compartment, as expected for endogenous heRF1 (Figure 4, upper panels). By contrast, in cells expressing NoLS-heRF1 (Figure 4, lower panels), nuclei were also decorated (Figure 4, merge panels). Furthermore, fluorescence was also associated with nuclear structures corresponding to nucleoli (Figure 4, arrows in lower panels). In conclusion, the 13-aa motif appears to be necessary and sufficient to import a cytoplasmic protein into the nucleus and to further drive it to the nucleolus. This motif, only present in the L-Ilf3 and L-NF90 isoforms, thus encompasses a dual NLS and NoLS.

### 4. Aminoacid deletion or substitution in the NoLS modulate the subnuclear distribution

**Figure 5:**
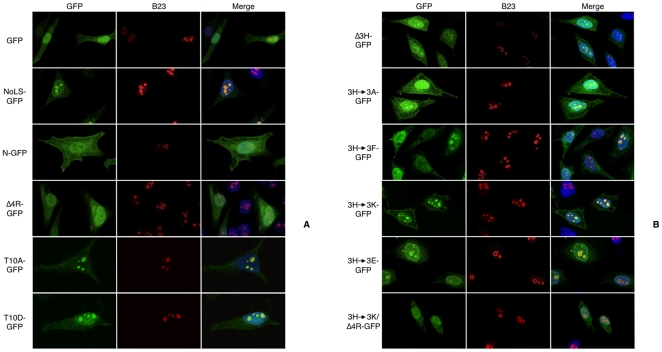
Subnuclear distribution of GFP fused to deletion or substitution mutants of Ilf3/NF90 NoLS. (**A**) Plasmids pEGFP-N1 (Control), pEGFP-N1-Ilf3/NF90 common N-terminal long region (NoLS-GFP), pEGFP-N1-Ilf3/NF90 common N-terminal short region (N-GFP), pEGP-N1/deletion mutant of the four-arginine stretch from the NoLS (Δ4R-GFP), pEGP-N1/substitution mutant of the threonine 10 by alanine (T10A-GFP) and pEGP-N1/substitution mutant of the threonine 10 by aspartate (T10D-GFP) and (**B**) plasmids pEGP-N1/deletion mutant of the three-histidine stretch (Δ3H-GFP), pEGP-N1/substitution mutant of the three-histidine stretch by three alanines (3H->3A-GFP), three phenylalanines (3H->3F-GFP), three lysines (3H->3K-GFP) or three glutamates (3H->3E-GFP) and pEGP-N1/substitution mutant of the three-histidine stretch by three lysines/deletion mutant of the four-arginine stretch (3H->3K/Δ4R-GFP) were transfected into HeLa cells. After 24 hours, cells were co-stained with the monoclonal anti-B23 antibody (B23) and DAPI. After confocal microscopy acquisition, focal planes exhibiting a B23 optimal signal were chosen. GFP fusion proteins appear in green, B23 in red and DAPI in blue.

To identify key residues for nucleolar targeting in the 13-aa sequence, deletion and substitution mutants were constructed using NoLS-GFP sequence (GFP coding sequence fused at the 3′ end of the sequence coding for the first 54 aa of the L-Ilf3/NF90 isoforms). Expression vectors were transfected into HeLa cells and after 24 hours cells were processed for indirect immunofluorescence with anti-B23 monoclonal antibodies. The intranuclear distribution of GFP and B23 was then observed by confocal microscopy (Figure 5). The three upper rows of figure 5A show the subcellular localization of GFP alone, wild-type NoLS-GFP and N-GFP (GFP coding sequence fused at the 3′ end of the sequence coding for the first 41 aa of the S-Ilf3/NF90 isoforms). While NoLS-GFP (positive control) accumulated in nucleoli, GFP alone and N-GFP (negative controls) uniformly labeled the whole nucleus and were seemingly excluded from nucleoli.

The 13-aa sequence contains a four-arginine stretch, which fits well with eukaryotic or viral NoLS consensus, K/R-K/R-X-K/R [28]–[30]. Deletion of the four arginines (Figure 5A, Δ4R-GFP row) led to the loss of nucleolar localization, as indicated by the absence of colocalization with B23.

As shown above, the 13-aa sequence can drive heRF1 into the nucleus (Figure 4). Given the similarity between the NoLS and basic NLS, one can ask why any NLS-containing protein is not automatically targeted to the nucleolus. What is required in addition to these four positively-charged residues to become an efficient and specific NoLS? Looking at the residues adjacent to the four-arginine stretch within the 13-aa sequence, two features could be singled out: a threonine residue predicted to be phosphorylated by protein kinase C and a three-histidine stretch.

When the threonine was replaced by alanine (T10A) or by the phosphomimetic aspartate (T10D), no difference with the positive control (Figure 5A, NoLS-GFP) was observed (Figure 5A, T10A-GFP and T10D-GFP rows), indicating that neither this threonine residue nor its putative phosphorylation are involved in NoLS function. When the three histidines were deleted (Figure 5B, Δ3H-GFP row) or replaced by three alanines (Figure 5B, 3H->3A-GFP row) or three phenylalanine (Figure 5B, 3H->3F-GFP row), an intermediate situation between the positive and negative controls was observed: nucleoli were less fluorescent and the nucleoplasm appeared more fluorescent than in the positive NoLS-GFP control. Fluorescence quantification indicated that, in the Δ3H-GFP, 3H->3A-GFP and 3H->3F-GFP experiments, nucleoli emitted only two-thirds of the GFP fluorescence recovered in the positive control (cells quantified: 39, 14, 21 and 32, respectively). These results indicate that the loss or the replacement of the three histidines by three alanines or phenylalanines significantly weakens the nucleolar targeting/retention.

To assess the role of positive charges carried by the three histidine residues, they were substituted by three lysines or glutamates (Figure 5B, 3H->3K-GFP and 3H->3E-GFP rows). With the positively-charged lysines, the fluorescence levels of nucleoli were similar to those of NoLS-GFP (96%, n = 20) whereas with the negatively-charged glutamates (n = 11), the nucleoli fluorescence levels were similar or weaker to those of Δ3H-GFP, 3H->3A-GFP and 3H->3F-GFP mutants. Finally, when a double 3H->3K/Δ4R-GFP mutant was tested, no nucleolar targeting was observed (Figure 5B, 3H->3K/Δ4R-GFP row) similarly to Δ4R-GFP mutant. Altogether, these results show that the three added lysines cannot substitute for the four missing arginines and strongly suggest that the positive charges of the three histidines may be important to retain the proteins in the nucleolus. The four arginines should then be required to drive a cytoplasmic protein into the nucleus and, from there, up to the nucleolus whereas the three histidines should increase its retention time in the nucleolus, maybe through specific interactions with nucleolar constituants.

### 5. Long Ilf3/NF90 isoforms are present in the granular component of the nucleolus

**Figure 6:**
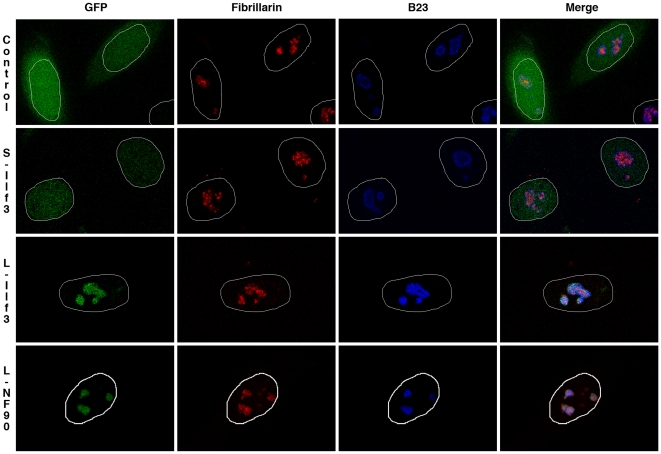
Subnuclear distribution of exogenously-expressed S-Ilf3, L-Ilf3 and L-NF90 isoforms in HeLa cells. Plasmids pEGFP-N1 (Control row), pEGFP-N1-S-Ilf3 (S-Ilf3 row), pEGFP-N1-L-Ilf3 (L-Ilf3 row) or pEGFP-N1-L-NF90 (L-NF90 row) were transfected into HeLa cells. After 24 hours, cells were co-stained with human anti-fibrillarin serum (Fibrillarin) and monoclonal anti-B23 antibody (B23). After confocal microscopy acquisition, focal planes were chosen to obtain optimal fibrillarin and B23 signals. DAPI (not shown here) was used to define the nuclear limits (white drawings). GFP and GFP fusion proteins appear in green, fibrillarin in red and B23 in blue.

To better define the subnucleolar localization of long isoforms, the GFP sequence was fused in frame after the S-Ilf3, L-Ilf3 or L-NF90 sequences. Constructs were transfected into HeLa cells and after 24 hours cells were processed for indirect double immunofluorescence with human anti-fibrillarin serum and anti-B23 monoclonal antibodies. The intranuclear distribution of GFP and markers was then observed by confocal microscopy (Figure 6).

In control cells expressing GFP alone as in cells transfected with the S-Ilf3 constructs, GFP fluorescence was observed in the whole nucleoplasm without any particular localization (Figure 6, Control and S-Ilf3 rows, left panels), whereas in cells transfected with the L-Ilf3 or L-NF90 constructs, GFP fluorescence was mostly restricted to the nucleoli (Figure 6, L-Ilf3 and L-NF90 rows, left panels). The fibrillarin and B23 antibodies decorating dense fibrillar and granular components, respectively (Figure 6, vertical middle panels) and the merged images showing a strict colocalization of L-Ilf3 and L-NF90 isoforms with B23 and not with fibrillarin (Figure 6, L-Ilf3 and L-NF90 rows, right panels), we concluded that the unmodified L-Ilf3 and L-NF90 isoforms appear to be mainly present in the nucleolus granular component [31].

### 6. Dynamic behavior of Ilf3 and NF90 during nucleolar disruption and reformation

DRB, an inhibitor of protein casein kinase II that phosphorylates nucleolar proteins, such as nucleophosmin, nucleolin and UBF, has been described to disorganize the nucleolus [32]. To analyze the behavior of Ilf3/NF90 during the reversible disruption and reformation of nucleoli, we have compared their dynamics with that of B23 during these processes.

**Figure 7:**
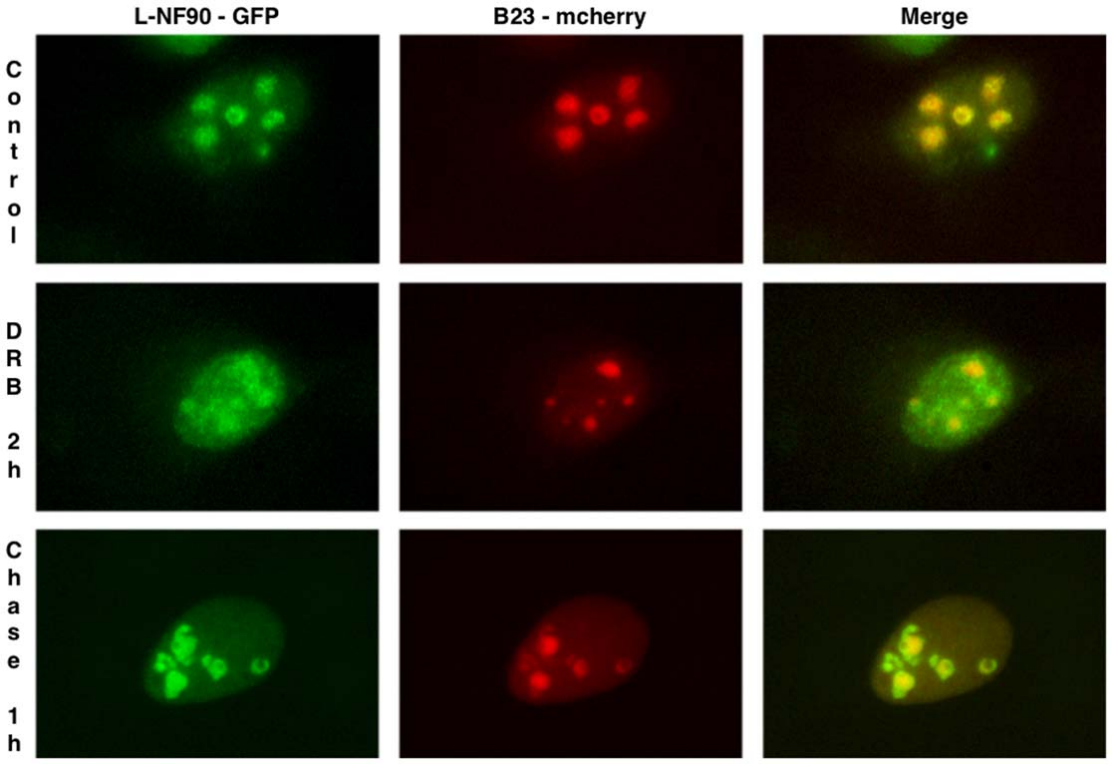
Subcellular distribution of exogenously-expressed L-NF90 isoforms in HeLa cells treated with DRB. Plasmids pEGFP-N1-L-NF90 (L-NF90-GFP) and mcherry-B23 (B23-mcherry) were transfected into HeLa cells. 24 hours later, cells were fixed either immediately (Control, upper panels) or after a DRB treatment during two hours (DRB 2 h, mid panels) followed by a chase of one hour (Chase 1 h, lower panels). GFP and mcherry fusion proteins appear in green and red, respectively.

When L-NF90-GFP and mcherry-B23 were co-expressed in HeLa cells, both proteins colocalized in the granular component (Figure 7, upper panels), as L-Ilf3 (Figure 5; lower panels). After treatment with DRB, the mcherry fluorescence was greatly reduced whereas the L-NF90-GFP fluorescence diffused in the nucleoplasm (Figure 7, middle panels). One hour after DRB removal, a partial restoration of nucleolar structures was observed. While B23 seemed to rapidly and almost totally localize into the reforming nucleoli, only a part of L-NF90 also relocalized with B23. The remaining L-NF90 was detected at the periphery of reforming nucleoli, around the B23 foci (Figure 7, lower panels). Identical results were obtained with L-Ilf3-GFP (data not shown).

### 7. L-Ilf3 and L-NF90 are highly dynamic and exchange between nucleoli

**Figure 8:**
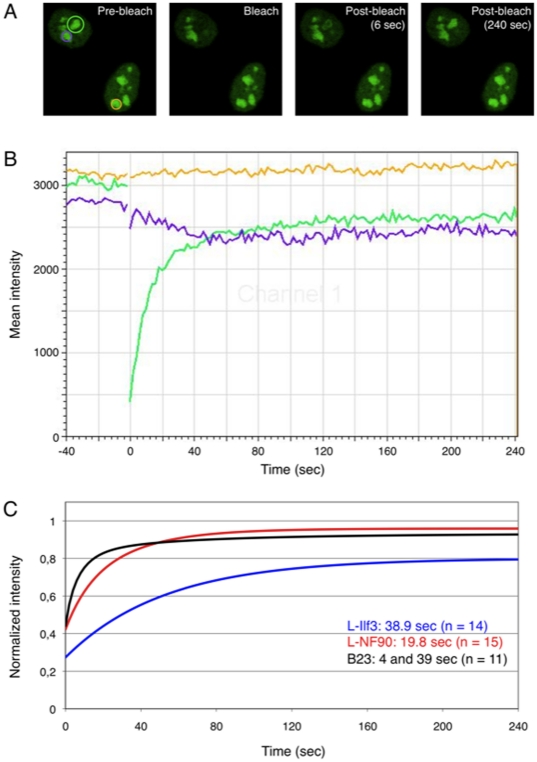
Fluorescence recovery in HeLa cell nucleoli after photobleaching (FRAP) of GFP-tagged L-Ilf3, L-NF90 or B23. Plasmids pEGFP-N1-L-Ilf3, pEGFP-N1-L-NF90 or pEGFP-N1-B23 were transfected into HeLa cells. After 24 hours, living cells were subjected to photobleaching. A. In HeLa cells expressing L-NF90-GFP, one nucleolus was targeted for laser bleaching (left panel, green circle in the upper cell) whereas another nucleolus from the same cell (blue circle in the upper cell) and a nucleolus from a distinct cell (yellow circle in the lower cell) were marked to serve as controls. Images were acquired every two seconds during 40 seconds before, immediately after or during 240 seconds after bleaching (prebleach, bleach, post-bleach 6 and 240 seconds, respectively). B. Fluorescence recordings emitted from the 3 delimited regions in A (green: bleached nucleolus; purple: control nucleolus from the same cell; yellow: control nucleolus from another unbleached cell). C. Kinetics of mean fluorescence recovery after photobleaching of GFP-tagged L-Ilf3 (n = 14), L-NF90 (n = 15) or B23 (n = 11) after a normalization of fluorescence intensity. The t^1^/_2_ of mean fluorescence recovery are indicated in the figure.

Knowing that L-Ilf3 and L-NF90 can dissociate from and reassociate with nucleoli (Figure 7), FRAP experiments using L-Ilf3-GFP, L-NF90-GFP and B23-GFP were performed to analyze precisely their kinetics. In HeLa cell nuclei, one nucleolus was photobleached then GFP fluorescence recovery was followed during 240 seconds, using one of the other non-bleached nucleoli of the same nucleus and one nucleolus of a neighboring cell as controls (Figure 8A).

Figure 8B shows GFP fluorescence recordings from the bleached nucleolus (green curve), from one adjacent nucleolus in the same nucleus (purple curve) and from one nucleolus of a distinct cell (yellow curve). The bleached nucleolus was rapidly re-colonized by L-NF90-GFP and its fluorescence level reached a plateau after 90 seconds post-bleach. At the same time, we observed that the fluorescence level of the adjacent nucleolus slightly and progressively decreased after the bleach and was stabilized after about 60 seconds. During this experiment, the fluorescence level of the nucleolus of the neighbor cell remained constant. These results indicate that L-NF90 behaves as a highly-dynamic protein which is rapidly exchanged between the different nucleoli within the same nucleus. Experiments performed with L-Ilf3-GFP and B23-GFP revealed the same dynamic exchange between nucleoli (data not shown), as reported also for several nucleolar proteins (38).

Computation of fluorescence recovery recordings from independent experiments with L-Ilf3-GFP (n = 14), L-NF90-GFP (n = 15) and B23-GFP (n = 11) is shown in figure 8C. t^1^/_2_ recovery, deduced for each protein (L-Ilf3: 38.9 seconds; L-NF90: 19.8 seconds; B23: 4 seconds and 39 seconds), indicated that L-Ilf3 and L-NF90 diffuse in a monodimensional manner whereas B23 appears to be composed of two mobile subpopulations, one diffusing more rapidly than the other, that is in opposition with previous report [33]. Finally, data analysis showed that L-Ilf3 mobile fraction represents 70% of total proteins, 93% for L-NF90 and 85% for B23.

Altogether, FRAP experiments indicate that L-Ilf3 and L-NF90 are each composed by a single population of highly mobile proteins that can exchange rapidly from one nucleolus to another within the same nucleus.

## Discussion

Previous studies showed that Ilf3 and NF90 are produced by alternative splicing in the 3′ region of their premessenger RNA [3], [4]. In addition, alternative splicing of exon 3 leads to the presence or absence of a 13-aa peptide at the N-terminus of both proteins [5]. Herein, we investigated in HeLa cells the influence of this sequence on the subcellular localization of recombinant fusion proteins (Figures 1, 4). We also analyzed the colocalization of GFP-tagged Ilf3/NF90 and B23 in HeLa cells and the distribution of endogenous Ilf3/NF90 in subcellular fractions of HeLa and P19 cells. Altogether, our results strongly suggest that the N-terminal 13 aa acts as a NoLS that also favors entry of the protein into the nucleus.

This NoLS contains three histidines followed by four arginines, forming a stretch of basic residues. Analysis of 61 previously-characterized or putative NoLS present in well-defined nucleolar proteins showed the presence of the nucleolar localization consensus motif, K/R-K/R-X-K/R [34], [35]. Many proteins are constantly or transiently associated with the nucleolus. Many of them contain a NoLS in which the consensus can be present in single or multiple copies that can be positioned anywhere in the primary sequence [28]-[30]. Surprisingly, computer analysis of these 61 NoLS showed that, among the eight possible combinations, the homogenous KKXK and RRXR motifs are found preferentially (30% and 40%, respectively; Table 1, bottom lane). Concerning the chemical nature of the residue X (Table 1, right column) and its observed frequency, as compared with that in vertebrate proteins, we noted a strong occurrence of proline (∼9.7% vs 5%), lysine (16.1% vs 7.2%), asparagine (17.7% vs 4.4%) and arginine (22.6% vs 4.2%) at this position. Acidic aspartate and glutamate were never found at this position, nor cysteine, tyrosine and histidine.

**Table 1:** Occurrence frequencies (%) of possible NoLS sequences (K/R-K/R-X-K/R) and of the twenty aminoacids at the X position in several NoLS-containing nucleolar proteins.

Motif	KKXK	KKXR	KRXK	KRXR	RRXR	RRXK	RXKR	RKXK	OF^a^	MF^b^	OF/MF
Res. X											
G	4.84	-	-	-	-	-	-	-	4.84	7.4	0.65
A	1.61	-	-	-	-	-	-	-	1.61	7.4	0.22
V	1.61	-	-	-	-	-	-	-	1.61	6.8	0.24
L	3.23	-	-	-	-	-	1.61	-	4.84	7.6	0.64
I	-	-	-	1.61	-	-	-	-	1.61	3.8	0.42
P	3.23	-	-	1.61	4.84	-	-	-	9.68	5.0	1.94
S	-	-	1.61	1.61	3.23	-	-	-	6.45	8.1	0.8
T	1.61	-	-	-	-	-	-	-	1.61	6.2	0.26
C	-	-	-	-	-	-	-	-	0.00	3.3	0.00
M	-	-	-	-	-	1.61	-	-	1.61	1.8	0.90
N	6.65	-	1.61	3.23	6.45	-	-	-	17.74	4.4	4.03
Q	-	1.61	-	-	3.23	-	-	-	4.84	3.7	1.31
F	-	-	-	-	1.61	1.61	-	-	3.23	4.0	0.81
Y	-	-	-	-	-	-	-	-	0.00	3.3	0.00
W	-	-	-	-	1.61	-	-	-	1.61	1.3	1.24
D	-	-	-	-	-	-	-	-	0.00	5.9	0.00
E	-	-	-	-	-	-	-	-	0.00	5.8	0.00
K	6.45	4.84	-	-	3.23	-	1.61	-	16.13	7.2	2.24
R	-	3.23	1.61	-	17.74	-	-	-	22.60	4.2	5.38
H	-	-	-	-	-	-	-	-	0.00	2.9	0.00
	29.03	9.68	4.84	8.06	41.93	3.23	3.23	0.00			
	51.61				48.39						

a Among the eight possible sequence combinations (upper line), observed frequency (OF) of each aminoacid at the X position in NoLS present in 61 well-characterized nucleolar proteins and using for the analysis.

b Mean frequency (MF) of each aminoacid observed in vertebrate proteins.

In the case of Ilf3 and NF90 L-isoforms, analysis of mutants indicated, in addition to the indispensable role of the four arginines to direct proteins to the nucleolus, the presence of positive charges close to the arginine stretch seems to be very important to retain these proteins in the nucleolus. The deletion or the replacement of the three histidines by uncharged (alanine or phenylalanine) or negatively-charged (glutamate) residues effectively decreases the efficiency of the nucleolar targeting by one third. *A contrario*, their substitution by three lysines with their strong and permanent positive charges maintains this efficiency.

Interestingly, whereas the distribution of endogenous Ilf3 in the nucleus revealed that most of Ilf3 was recovered in the nucleoplasm (Figures 2 and 3), the overexpression of L-Ilf3 showed a preferential localization in nucleoli (Figure 6). This observation could be explained by the fact that the unmodified L-Ilf3 isoforms are only recovered in the nucleoli while the posttranslationally-modified isoforms are in the nucleoplasm (Figure 3). Indeed, if we consider that overexpressed L-Ilf3 is not posttranslationally-modified because the modification enzymes are titrated by the excess of substrate proteins, we can explain why the majority of the chimeric L-Ilf3-GFP is associated with the nucleoli (Figure 6) and not found in the nucleoplasm, as endogenous L-Ilf3 (Figures 2, 3). Nuclear fraction of endogenous L-NF90 being exclusively associated with the nucleoli (Figure 2), independently of their posttranslational modifications status (Figure 3), the overexpressed L-NF90 is also only localized in the nucleoli (Figure 6). These observations could explain why a L-Ilf3 or L-NF90 overexpression leads to a cellular death (data not shown), certainly due to a perturbation of the nucleolar organization and functions resulting from an overaccumulation of overexpressed proteins.

The nucleolar localization of L-Ilf3/NF90 isoforms resembles that of proteins involved in nucleocytoplasmic transport of mRNA [36] or mRNA editing [37]. For these proteins, it was suggested that they assemble with their cargo mRNA into RNP directly in the nucleolus or first in the nucleoplasm and then localize to the nucleolus [38]. In both cases, RNP particles leave the nucleolus after an eventual binding of nucleolar proteins and/or RNAs and are then exported to the cytoplasm. The functional reason for re-routing RNP particles *via* the nucleolus on the way to the cytoplasm is not clear, but two hypotheses can be considered. Either the passage by the nucleolus is a checkpoint to ensure the functional integrity of RNP complexes, or it represents a sequestration step allowing some negative regulation to occur on mRNA translation and/or protein activity [38]. Whatever it may be, since Ilf3/NF90 have been previously reported to interact with several RNAs [1], [15]-[20], the idea of a transient association of RNP containing Ilf3/NF90 with the nucleolus was reinforced by data showing that they interact with the nucleolar protein ADAR [39].

At the end of mitosis or after a DRB treatment, the timing of incoming proteins in the reassembling nucleoli depends on their subnucleolar localization, hence on their function [31], [32], [40]. Indeed, the proteins recovered in the fibrillar component and implied in early rRNA processing steps, e.g., fibrillarin and UBF, arrived at first whereas the proteins of the granular component responsible for late rRNA processing stages, e.g., B23 and Nop52, relocalized to the nucleolus later [31], [32], [40]. Even if some L-NF90 isoforms can reintegrate rapidly the granular component, the relocalization of most of them occurs much later. This difference in the timing of the relocalization of L-NF90 and the other granular component proteins would suggest that L-NF90 (and certainly unmodified L-Ilf3 also present there) could be implicated either in the very last stages of rRNA processing and/or preribosomes assembly or in other yet unknown functions.

FRAP experiments have shown that L-Ilf3 and L-NF90 exhibit a highly dynamic behavior and can exchange between different nucleoli within the same nucleus. This corresponds to a typical behavior for numerous nucleolar proteins [31]. These internucleolar exchanges indicate that all the nucleoli of a given nucleus can be considered as a single nuclear compartment instead of independent components. In the FRAP experiments described in this paper, only the fluorescence recovery of L-Ilf3-GFP and L-NF90-GFP present in nucleoli is measured, assuming that the endogenous proteins behave similarly. The t^1^/_2_ recovery values corresponding to nucleoplasmic or cytoplasmic Ilf3/NF90 may be different from those reported here.

Herein, we showed that the N-terminal 13-aa sequence of the L-Ilf3 and L-NF90 proteins, encoded by the alternatively-spliced exon 3, allows their subcellular localization to nucleoli, thus acting as a NoLS. Moreover, this particular sequence is necessary and sufficient to target unrelated cytoplasmic polypeptides to the nucleolus.

Considering the numerous cellular functions previously reported for Ilf3 and NF90 [1], [6], [7], [11]-[24] and their multiple cellular localizations ([1], [23], [26], [27]; our results), and given that all isoforms are not present in all cellular compartments, each isoform may have specialized functions. Moreover, taking into consideration the important polymorphism of Ilf3/NF90, it is obvious that this protein heterogeneity is not generated solely by alternative splicing events, but also by posttranslational modifications, such as arginine methylation [6] and phosphorylation [4], [7], [8]. Analyses of their implication in the subcellular localization of Ilf3/NF90 and/or in the regulation of their functions are currently in progress.

## Materials and Methods

### Cell culture

HeLa and P19 cells were cultured as described [5]. When mentioned, cultured cells were treated for two hours by addition of 5,6-dichloro-1-ß-D-ribofuranosylbenzimidazole (DRB; Sigma-Aldrich, St Louis, MO, USA) at the concentration of 240 µmole.L^-1^.

### Indirect immunofluorescence

HeLa cells were fixed as described [1]. Subsequent steps were performed in PBS supplemented with 0.2% (v/v) Triton X100 and 1% (w/v) bovine serum albumine. Cells were stained for one hour at room temperature with monoclonal anti-α-tubulin DM1A (1∶1000; Sigma-Aldrich), polyclonal anti-heRF1 antibodies (1∶200), human polyclonal anti-fibrillarin D157 serum (1∶500) and/or monoclonal anti-B23 antibody FC82291 (1∶500; Sigma-Aldrich). Primary antibodies were revealed using the Alexa Fluor 488 goat anti-rabbit (1∶1000; Molecular Probes, Eugene, OR, USA), the Alexa Fluor 660 goat anti-human (1∶1000; Invitrogen) or the Alexa Fluor 660 goat anti-mouse Igs (1∶1000; Invitrogen). Each incubation was followed by three PBS washes and coverslips were mounted onto glass slides in Citifluor™ (Citifluor Ltd, Leicester, UK). When needed, cells were incubated 10 minutes with PBS containing 4′,6-diamidino-2-phenylindole (DAPI, 0.1 µg.mL^−1^) to visualize DNA and rinsed twice with PBS before mounting.

### Subcellular fractionation

Subcellular fractionation of HeLa and P19 cells was performed by differential centrifugation as described [41] with modifications in the buffer A composition (Tris-HCl 10 mmol.L^−1^ pH 7.4, KCl 10 mmol.L^−1^, MgCl_2_ 1.5 mmol.L^−1^, dithiothreitol 0.5 mmol.L^−1^). Protein concentrations were determined using the Micro BCA Protein Assay Reagent Kit (Pierce, Rockford, IL, USA).

### 1D- and 2D-PAGE and Western blot

1D- and 2D-PAGE were performed as described [42], [43] with minor modifications [44]. Electrotransfer of proteins onto nitrocellulose (Hybond C, GE Healthcare, Chalfont St Giles, UK) was performed according to [45] with minor modifications [44]. After membrane saturation [1], polyclonal anti-Ilf3/NF90 serum (Ab78; [1]), monoclonal anti-Ilf3/NF90 antibodies (BD Biosciences, Palo Alto, CA, USA), monoclonal anti-α-tubulin antibodies (DM1A; Sigma-Aldrich), monoclonal anti-B23 antibodies (B0556; Sigma-Aldrich) or human polyclonal anti-UBF D165 serum were incubated overnight at room temperature and revealed with peroxidase-linked secondary antibodies (Sigma-Aldrich) by the chemiluminescence method.

### Plasmid constructions

Numbering of Ilf3 and NF90 primer oligodeoxynucleotides (MWG, Courtabœuf, France; Eurogentec, Liège, Belgium) was done according to the mouse Ilf3 and NF90 sequence published previously (GenBank accession numbers AF447751/NM010561, AF447752/NM001042707, DQ104405/NM001042708 and DQ104406/NM001042709; 5), italic types correspond to non-complementary sequences used to introduce specific restriction sites (underlined characters, *AGATCT*: *Bgl* II and *GAATTC*: *Eco*R I) or the Kosak consensus sequence (double-underlined characters, *GCCACC*), respectively.

To construct the short or long Ilf3/NF90 N-terminus in frame with the GFP (Short- or N- and Long- or NoLS-GFP), PCR were performed using plasmids pSK^+^ containing Ilf3 as template (5) and IN-51 (193-236: 5′-*CCGGAATTCGCCACC* **ATG**GCATTGTATCATCATCACTTCATCACAAGAAGAGAAGGCG-3′) or IN-52 (193-229: 5′-*CCGGAATTCGCCACC* **ATG**CGTCCCATGAGAATTTTTGTGAATGATGATCGCC-3′) as 5′ primer and IN-31 (528−500 or 489−461: 5′-GGCTACCAGGCCGACCCGCATCACGCCCC-3′) as 3′ primer. Final PCR products were digested with *Eco*R I and *Apa* I, isolated by migration in an 2.0% (w/v) agarose gel, electroeluted and finally subcloned in pEGFP-N1 (BD Biosciences) previously linearized with the same enzymes.

To construct the short or long N-terminus Ilf3/NF90 in frame with human eRF1 (N-heRF1 and NoLS-heRF1), PCR were performed using Long-GFP and Short-GFP plasmids as template and IN-53 (193-197: 5′-*GCTCAAGCTTCGAATTCGCCACC* **ATG**GC-3′) or IN-54 (193-197: 5′-*GCTCAAGCTTCGAATTCGCCACC* **ATG**CG-3′) as 5′ primer and IN-32 (355-338 or 316-299: 5′-*CATCCAGATCT*GGGCCCGCTCAGTATGGG-3′) as 3′ primer. PCR products were digested with *Eco*R I and *Bgl* II, isolated by migration in an 1.5% (w/v) agarose gel, electroeluted and finally subcloned in pCMV-heRF1 [45] previously linearized with the same enzymes.

To construct the deletion and substitution NoLS-GFP mutants, site-directed mutagenesis PCR were performed using NoLS-GFP plasmid as template and primers containing some non-complementary nucleotides allowing to introduce mutation codons (bold types). To construct Δ4R-GFP mutant, Δ4R-5 (5′-ATGGCATTGTATCATCACTTCATCACACGTCCCATGAGAATTTTTGAATGATGATCG-3′) and Δ4R-3 (5′-CGATCATTCAAAAATTCTCATGGGACGTGTGATGAAGTGATGATGATACAATGCCAT-3′) were used as 5′ and 3′ primers, respectively. To construct T10A-GFP and T10D-GFP mutants, T10A-5 (5′-GCATTGTATCATCATCACTTCATC**GCA**AGAAGAAGAAGG-3′) or T10D-5 (5′-GCATTGTATCATCATCACTTCATC**GAT**AGAAGAAGAAGG-3′) and T10A-3 (5′-CCTTCTTCTTCT**TGC**GATGAAGTGATGATGATACAATGC-3′) or T10D-3 (5′-CCTTCTTCTTCT**ATC**GATGAAGTGATGATGATACAATGC-3′) were used as 5′ and 3′ primers, respectively. To construct Δ3H-GFP, 3H->3A-GFP, 3H->3E-GFP, 3H->3F-GFP and 3H->3K-GFP mutants, Δ3H-5 (5′-CCGGAATTCGCCACCATGGCATTGTATTTCATCACAAGAAGAAGAAGGCGTCCC-3′) or 3H->3A-5 (5′-CCGGAATTCGCCACCATGGCATTGTAT**GCTGCAGCG**TTCATCACAAGAAGAAGAAGGCGTCCC-3′) or 3H->3E-5 (5′-CCGGAATTCGCCACCATGGCATTGTAT**GAGGAAGAG**TTCATCACAAGAAGAAGAAGGCGTCCC-3′) or 3H->3F-5 (5′-CCGGAATTCGCCACCATGGCATTGTAT**TTCTTCTTC**TTCATCACAAGAAGAAGAAGGCGTCCC-3′) or 3H->3K-5 (5′-CCGGAATTCGCCACCATGGCATTGTAT**AAGAAGAAA**TTCATCACAAGAAGAAGAAGGCGT-3′) and Δ3H-3 (5′-CGCCTTCTTCTTCTTGTGATGAAATACAATGCCATGGTGGCGAATTC-3′) or 3H->3A-3 (5′-CGCCTTCTTCTTCTTGTGATGAA**CGCTGCAGC**ATACAATGCCATGGTGGCGAATTC-3′) or 3H->3E-3 (5′-CGCCTTCTTCTTCTTGTGATGAA**CTCTTCCTC**ATACAATGCCATGGTGGCGAATTC-3′) or 3H->3F-3 (5′-CGCCTTCTTCTTCTTGTGATGAA**GAAGAAGAA**ATACAATGCCATGGTGGCGAATTC-3′) or 3H->3K-3 (5′-CGCCTTCTTCTTCTTGTGATGAA**TTTCTTCTT**ATACAATGCCATGGTGGCGAATTC-3′) were used as 5′ and 3′ primers, respectively. To construct the 3H->3K/Δ4R-GFP double-mutant, site-directed mutagenesis PCR were performed using 3H->3K-GFP (see above) as template, and 3H->3K/Δ4R5 (5′-CCGGAATTCGCCACCATGGCATTGTATAAGAAGAAATTCATCACACGTCCCATGAG-3′) and 3H->3K/Δ4R3 (5′-CTCATGGGACGTGTGATGAATTTCTTCTTATACAATGCCATGGTGGCGAATTC-3′) as 5′ and 3′ primers, respectively. All products resulting from site-directed mutagenesis PCR were digested with *Dpn* I to discard methylated DNA template before transformation in bacteria.

To amplify expression vectors, constructs were introduced in *Escherichia coli* strain XL1 blue (Stratagene, Santa Clara, CA, USA) then plasmids were purified using the QIAfilter Plasmid Midi Kit (Qiagen, Mayence, Germany).

### DNA transfection

DNA transfection in HeLa and P19 cells was performed by electroporation as described [46] or using Nanofectin reagent (PAA, Pasching, Austria). After 24 hours of culture, HeLa cells were fixed then used either for direct GFP fluorescence or indirect immunofluorescence analysis [1]. Indirect immunofluorescence was performed with monoclonal anti-B23 antibody FC82291 (Sigma-Aldrich), anti-fibrillarin D157 human polyclonal serum (Dr Danièle Hernandez-Verdun' gift), rabbit serum S19 that recognizes eRF1 [46] and anti-α-tubulin DM1A monoclonal antibody (Sigma-Aldrich).

### Widefield microscopy

The different slides were observed using an upright BX41 Olympus microscope with 60X (1.25 N.A.) UPlan F1 APO objective. The DAPI was visualized with the DAPI/Hoechst/AMCA filter cube (Exc. 325-375, dic. 400, em. 435–485; Chroma Technology Corporation, Bellows Falls, VT, USA), the GFP or Alexa 488 fluorescence were detected with FITC filter cube (Exc. 465–495, dic. 505, em. 515–555; Chroma Technology Corporation, Bellows Falls, VT, USA), the Alexa 568 fluorescence was detected with mcherry filter cube (Exc. 530–560, dic. 570, em. 572–648; Chroma Technology Corporation, Bellows Falls, VT, USA) and the filter cube Y5 (exc. 630–650, dic. 660, em. 665–695; Chroma Technology Corporation, Bellows Falls, VT, USA) was used for visualization of Alexa 660. The different images were acquired using the CoolSnap Cf CCD camera (Photometrics, Roper Scientific, Tucson, AZ, USA) driven by Metamorph software (Molecular Devices, Sunnyvale, CA, USA).

### Confocal microscopy

The cells were imaged using confocal laser scanning microscopy (Leica SP2 or Leica SP5 system, Leica Microsystems, Heidelberg, Germany) using a HCX PL APO CS 63.0X 1.4NA oil UV objective. DAPI, GFP or Alexa 488, Alexa 568 and Alexa 660 were excited sequentially with 405, 488, 561 and 633 nm laser lines, respectively and the fluorescence were selected between 415–460, 500–535, 550–620 and 640–735 nm, respectively. The overlay images were done with ImageJ software.

### Photobleaching and live cell microscopy

24 hours after transfection, pre-warmed culture medium without phenol red and supplemented with fetal calf serum (10%, v/v) and Hepes (2 mmol.L^−1^ pH 8.0) was added to HeLa cells. Coverslips were mounted in a Ludin chamber (Life Imaging Service, Basel, Switzerland) and cells were imaged with a Leica TCS-SP5 confocal laser scanning microscope (Leica, Solms, Germany) with a 63X 1.4NA oil UV objective. During experiments, cells were kept at 37°C using air conditioning chamber (Life Imaging Service). The regions of interest (ROI) were bleached with the 488 nm laser at full power whereas imaging acquisitions were done at 4% of laser full power. Images were collected in 512×512 format using the Leica LAS-AF software every two seconds during 40 seconds before, immediately after and at two seconds intervals after nucleolar bleaching for 240 seconds.

### Quantification of relative fluorescence intensity

Fluorescence intensity was measured using Image J 1.4g software. For FRAP experiments, the average intensity in the ROI before bleaching, immediately after bleaching and post-bleaching was measured. Fluorescence intensity of the nucleus was also measured. Background fluorescence was measured in a field outside the cell and subtracted from the nucleolar and nuclear fluorescence values. The normalized fluorescence intensity (FI) was calculated as follows: FI =  (INO_t_/IN_t_)/(INO_0_/IN_0_), where INO_t_ corresponds to the average fluorescence intensity of the photobleached nucleolus at various time points after photobleaching, IN_t_ to the average fluorescence intensity of the entire nucleus at the corresponding time point, INO_0_ to the average fluorescence intensity of the photobleached nucleolus before photobleaching and IN_0_ to the average fluorescence intensity of the entire nucleus before photobleaching.

Non-linear curve fitting of the recovery data was carried out with Microsoft Excel using the “Solver” macro. The fluorescence recovery was plotted against time and t^1^/_2_ recovery was calculated using the following equations depending whether the diffusion corresponds to mono- or biexponential type: F(t)  =  ((F_inf_ −F_0_). (1−e^(−t/t1/2)^) +F_0_) or F(t)  =  ((F_inf_−F_0_). (((1−A). e^(−t/t1/2,1)^) − ((1−A). e^(−t/t1/2,2)^)) +F_0_) where F_0_ corresponds to the intensity value immediately after bleaching, F_inf_ to the intensity value at the end of the experiment, t to the time point and A to one recovery fraction. All parameters were determined from the fitting of each mean curve. The total mobile and the total immobile fractions were calculated on the fitted mean curves as described [47]. The total mobile fraction (MF) is given by (F_inf_−F_0_) / (1−F_0_) and the immobile fraction (IF) by 1−MF.
